# Cognitive Function Mediates the Association Between Type 2 Diabetes and Physical Performance Decline in Older Adults: A Cross-Sectional Study

**DOI:** 10.3390/healthcare14162581

**Published:** 2026-08-17

**Authors:** Xiaohong Chen, Zheping Zhou, Yuchen Ma, Zongyi Chen, Honghong Zhang, Yueju Wang, Ji Hu

**Affiliations:** 1 Department of Endocrinology and Metabolic Medicine, The Second Affiliated Hospital of Soochow University, Suzhou 215006, China; 2 Department of Geratology, The First Affiliated Hospital of Soochow University, Suzhou 215006, China; 3 Department of Geratology, Afiliated Changshu Hospital of Nantong University, Changshu 215500, China

**Keywords:** type 2 diabetes mellitus, cognitive impairment, physical performance, gait speed, grip strength, mediation analysis, cross-sectional study, older adults

## Abstract

**Background:** Elderly patients with type 2 diabetes mellitus (T2DM) often experience cognitive decline and physical function degradation. However, whether cognitive function mediates the association between T2DM and physical performance in older adults has not been previously examined. This study aimed to explore whether cognitive function was statistically associated with the relationship between T2DM and physical performance in older adults using mediation analysis. **Methods:** We conducted a cross-sectional study, and a total of 102 participants aged ≥60 were included (46 patients with T2DM and 56 non-diabetes controls). Cognition was assessed using standardized neuropsychological tests (global cognition, as assessed by the MoCA total score; and domain-specific measures). Physical performance was quantified by walking speed and handgrip strength. Mediation analyses were performed to test whether the cognitive function statistically accounted for part of the association between the T2DM state and the physical performance. **Results:** Participants with T2DM had poorer cognitive performance and lower physical performance than controls. In mediation models, the MoCA total score statistically accounted for approximately 18% of the association between T2DM and gait speed (ACME −5.19 cm/s, 95% CI −8.73 to −2.32; proportion mediated 0.18, 95% CI 0.08–0.32). For grip strength, the corresponding proportion was estimated at 60% (ACME −2.16 kg, 95% CI −3.78 to −0.81; proportion mediated 0.60, 95% CI 0.23–1.81), although this estimate should be interpreted cautiously because of the wide confidence interval. **Conclusions:** This study suggests that poorer cognitive performance may partially account for the observed relationship between T2DM and the decline in physical performance of the elderly. The results support the presence of concurrent cognitive and physical impairment among older adults with T2DM. Given the cross-sectional design, these findings should be regarded as exploratory and hypothesis-generating, and longitudinal studies are required to establish temporal relationships.

## 1. Introduction

Type 2 diabetes mellitus (T2DM) is one of the most pressing global health challenges of the 21st century, and its prevalence among the elderly population is rising dramatically. In addition to well-known complications such as peripheral neuropathy and microvascular disease, more and more evidence has linked T2DM with accelerated decline in cognitive function and premature degradation of physical function [1,2]. These effects can have a far-reaching impact on the elderly, threatening the independence of their daily activities, increasing the probability of falls and fractures, and ultimately increasing the risk of being admitted to medical institutions and death [3]. Therefore, the simultaneous decline of cognition and physical decline in diabetes has put a heavy burden on patients themselves, their families and the global health system.

T2DM is associated with a significantly increased risk of cognitive decline and dementia [4]. These impairments typically affect episodic memory, executive function, processing speed, animal fluency, and clock drawing, ultimately impairing daily functioning [5]. The underlying mechanisms mainly include cerebral vascular injury induced by chronic hyperglycemia, blood–brain barrier dysfunction, neuroinflammation, brain insulin resistance and the accumulation of advanced glycosylation end products [6,7]. Neuroimaging studies further show hippocampal atrophy, white matter hyperintensities, and decreased cerebral blood flow [8]. Despite these findings, the exact pathophysiological mechanisms require further investigation.

In parallel with cognitive degeneration, elderly patients with T2DM will also have a significant decline in physical performance beyond traditional complications [9]. As a strong indicator of overall health, walking speed has been proven to be significantly reduced in diabetic individuals, and some studies have reported that it has decreased by 10–30% compared with age-matched controls [10,11]. At the same time, grip strength is a sign of overall muscle strength and a predictor of functional limitation. Even after adjusting for the age and body composition, the grip strength of diabetic populations tends to be lower [12]. These defects can translate into foreseeable consequences: slower pace is associated with a higher risk of falling, hospitalization, loss of independence and death, while reduced grip strength can predict disability and frailty [13,14]. The mechanism of physical function decline is multifaceted, including diabetic peripheral neuropathy, accelerated sarcopenia, microvascular lesions affecting muscle perfusion, chronic systemic inflammation and skeletal muscle mitochondrial dysfunction. It is worth noting that these damages often occur early in the disease and may progress insidiously. Taken together, these diabetes-related neural and musculoskeletal pathologies may co-occur and interact, suggesting that cognitive decline could be linked to physical performance deterioration in older adults with T2DM. Whether variation in cognitive performance is statistically associated with the relationship between T2DM and physical performance, however, remains unclear.

Although the association between T2DM and cognitive and physical decline has been fully documented, these two areas were still studied separately. Existing studies mostly examine cognitive decline or physical performance defects as outcomes, ignoring the possible interrelationship between the two. A few studies on the cognitive–physical relationship were mainly carried out in the general aging population, but not in T2DM where both domains may be simultaneously affected [15]. More importantly, although cognitive impairment and physical dysfunction frequently coexist in T2DM, it remains unclear whether cognitive performance statistically accounts for part of their observed association. Given that cognitive function is crucial for planning and executing complex motor tasks, maintaining balance, and regulating gait patterns [16]. Addressing this gap may improve risk stratification and support the development of integrated intervention strategies. If poorer cognitive performance is statistically associated with physical performance deficits among older adults with T2DM, cognitive assessment may help identify individuals at greater risk of functional decline and support more comprehensive management strategies.

In order to make up for these knowledge gaps, this study comprehensively examined the relationship between cognitive function and physical performance of the older adults with or without T2DM recruited from the elderly outpatient clinic. Our main objectives were to compare the differences between elderly patients with T2DM and age-matched controls in terms of cognitive function and physical performance indicators; to investigate the correlation between cognitive function and physical performance in each group; and to conduct a statistical mediation analysis to explore whether cognitive function statistically accounted for part of the association between T2DM status and physical performance deficits. This research may lead to shifting the focus to earlier cognitive assessment and multi-field comprehensive intervention in diabetic populations.

## 2. Materials and Methods

### 2.1. Study Design and Participants

This cross-sectional study was carried out in the Geriatrics Clinic of the First Affiliated Hospital of Soochow University. Eligible participants were consecutively recruited during routine outpatient visits. The study protocol was approved by the Ethics Committee of Soochow University (Approval No. SUDA20241021A01), and written informed consent was obtained from all participants and/or their caregivers.

Participants were included if they were 60 years or older, could walk independently for at least 10 min without a walking aid, and had no severe acute or terminal illness that would prevent safe cognitive or physical performance testing. Type 2 diabetes mellitus (T2DM) was diagnosed by an endocrinologist according to American Diabetes Association criteria based on outpatient medical records, and all diagnoses were checked by a second reviewer. For patients with T2DM, we additionally required a documented physician diagnosis, no acute hyperglycemic crises or severe hypoglycemia requiring medical intervention in the past 3 months, and stable antidiabetic treatment without changes in oral agents or insulin in the previous 3 months. The control group consisted of non-diabetic outpatients attending the same Geriatrics Clinic during the same study period.

We excluded participants with neuromuscular or musculoskeletal diseases that could markedly affect mobility or physical performance, such as stroke, Parkinson’s disease, severe osteoarthritis, major lower-limb trauma, or lower-limb surgery. We also excluded those who could not cooperate with neuropsychological or physical performance tests because of mental disorders or severe cognitive or behavioral symptoms; those with severe visual or hearing impairment that could interfere with test administration or understanding, including moderate to severe non-proliferative or proliferative diabetic retinopathy confirmed by ophthalmologic examination; and those with cardiovascular, peripheral vascular, or respiratory diseases that limited walking or exertion, such as a history of myocardial infarction, heart failure (NYHA class III–IV), unstable angina, or clinically significant arrhythmias.

Use of drugs known to substantially affect cognition or motor performance within 14 days before assessment was another exclusion criterion. These drugs included benzodiazepines, antipsychotics, tricyclic antidepressants or other strongly anticholinergic agents, opioid analgesics, skeletal muscle relaxants, and sedative-hypnotics. We also excluded patients with severe diabetic complications that could directly impair physical performance, such as active diabetic foot ulcers or Charcot neuroarthropathy, and those with other acute or unstable medical conditions that might change mobility, gait stability, or test performance (for example, acute infection, acute vertigo, severe pain, orthostatic hypotension with symptoms, unplanned hospitalization, or major treatment changes in the previous 2 weeks). A documented fall in the last 3 months that led to a persistent change in walking pattern or mobility was also an exclusion criterion. Participants with previously diagnosed dementia or mild cognitive impairment were not excluded, provided they were able to complete all study assessments. All participants completed standardized medical history taking, a comprehensive neuropsychological assessment, and evaluation of daily living abilities and physical performance (habitual gait speed and handgrip strength).

### 2.2. Sociodemographic Characteristics and Cognitive Assessment

Sociodemographic data (age, sex, height, weight, years of education, and medical history) were collected in face-to-face interviews using a structured questionnaire (Years of education were defined as the total number of years of formal schooling starting from primary school). Body mass index (BMI) was calculated as weight (kg) divided by height squared (m^2^). Hypertension and other chronic diseases were determined from medical records and physician diagnoses.

Global cognition was assessed using the Mini-Mental State Examination (MMSE) and the Beijing version of the Montreal Cognitive Assessment (MoCA-BJ) [17]. One additional point was added to the MoCA-BJ score for participants with fewer than 12 years of education [18]. The Clock Drawing Test (CDT) was used to evaluate executive and visuospatial function [19]. Memory was tested with the Chinese version of the Auditory Verbal Learning Test (AVLT), including immediate recall, delayed recall, and recognition [20]. Semantic fluency was assessed with the animal-naming Verbal Fluency Test [21]. Affective symptoms were assessed using the Hamilton Anxiety Rating Scale (HAMA) and the Hamilton Depression Rating Scale (HAMD) at the study visit [22]. Instrumental activities of daily living were evaluated with the Instrumental Activities of Daily Living Scale (IADL) [23]. All cognitive tests were administered by trained physicians from the Neurology team of the Department of Geriatrics, following standardized procedures. Assessors were not informed of participants’ T2DM status during test administration and scoring. Physical performance assessments were conducted by a geriatrician and an endocrinologist using standardized procedures.

### 2.3. Physical Performance: Gait Speed and Handgrip Strength

Habitual gait speed was measured on a 7 m straight walkway marked at the start and end. Participants wore comfortable shoes and were asked to walk at their usual pace. Gait speed was assessed twice at usual pace and averaged after one practice trial. Gait speed parameters were recorded using a wireless inertial-sensor motion analysis system (Mobility Lab™, APDM Inc., Portland, OR, USA) [24].

Handgrip strength was measured with a calibrated handheld dynamometer. Participants were seated with the shoulder adducted and in a neutral position, the elbow flexed at about 90°, and the forearm in a neutral position. They were asked to squeeze the dynamometer with maximum effort for 3 s. Two trials were performed for each hand with short rests between trials, and the highest value (in kilograms) from either hand was used in the analyses.

### 2.4. Statistical Analysis

All analyses were performed using R software (version 4.5.1). Continuous variables were first examined for distributional characteristics using the Shapiro–Wilk test and visual inspection of Q–Q plots. Variables that approximated a normal distribution (based on the Shapiro–Wilk test and Q–Q plots) were compared between groups using independent-samples *t* tests; otherwise, the Mann–Whitney U test was applied. The false discovery rate (FDR) was controlled for using the Benjamini–Hochberg procedure for multiple between-group comparisons across cognitive outcomes. Correlation analyses were prespecified as exploratory analyses aimed at describing associations within the T2DM group; therefore, nominal *p* values are reported without additional multiplicity adjustment. Continuous variables are presented as mean ± standard deviation (SD) for approximately normally distributed variables and as median (interquartile range, IQR) otherwise and categorical variables as counts and percentages. Effect sizes for between-group differences in cognitive outcomes were calculated using Hedges’ g (≈0.2 is small, ≈0.5 is moderate, ≈0.8 is large).

Associations between cognitive function and physical performance were examined using Pearson correlation coefficients after checking linearity assumptions. Correlation results are presented in both tabular form and as a heat map to highlight the cognitive domains most strongly related to gait speed and grip strength.

To evaluate whether global cognitive function mediates the relationship between T2DM and physical performance, mediation analyses were performed separately for gait speed and grip strength using the “mediation” package in R [25]. T2DM status (T2DM vs. control) was specified as the exposure, MoCA total score as the mediator, and gait speed (cm/s) or grip strength (kg) as the outcome. Both mediator and outcome models were adjusted for age, sex, years of education, body mass index (BMI), and hypertension. The average statistical mediation effect (ACME, indirect effect), average direct effect (ADE), total effect, and proportion mediated were estimated with 5000 nonparametric bootstrap resamples to obtain 95% confidence intervals, as a prespecified and pragmatic balance between interval stability and computational burden for this exploratory analysis. These mediation models assume (i) no unmeasured confounding of the exposure–mediator, mediator–outcome, and exposure–outcome relationships conditional on covariates, (ii) correct model specification, and (iii) no exposure–mediator interaction unless modeled. Linearity and model specification were evaluated by inspection of residual plots and diagnostic statistics. No exposure–mediator interaction was specified because the primary objective was to estimate the average indirect effect under the standard mediation framework. Given the cross-sectional measurement, mediation findings are interpreted as exploratory and do not establish temporal precedence.

As a sensitivity analysis, the mediation models were repeated using minimally adjusted models including age and sex only (Supplementary Table S1). In addition, sensitivity analyses for potential unmeasured mediator–outcome confounding were performed using the medsens function in the R mediation package, and the robustness of the estimated indirect effects was evaluated across different values of the residual correlation parameter (ρ) (Supplementary Figures S1 and S2).

## 3. Results

### 3.1. Participant Characteristics

A total of 102 participants were included in the analysis, comprising 46 individuals with T2DM and 56 non-diabetic controls. As shown in Table 1, the two groups did not differ significantly in age (mean ± SD: 74.72 ± 8.04 vs. 73.48 ± 7.47 years, *p* = 0.427), BMI (median (IQR): 22.50 (20.25, 24.70) vs. 22.45 (20.25, 24.80) kg/m^2^, *p* = 0.649), sex distribution (*p* = 0.255), or prevalence of hypertension (*p* = 0.092). Participants with T2DM had fewer years of education than controls (mean ± SD: 7.87 ± 5.26 vs. 9.91 ± 4.19 years, *p* = 0.036). Both education and hypertension were adjusted for in subsequent mediation analyses.

### 3.2. Group Differences in Cognitive Function and Physical Performance

**Figure 1:**
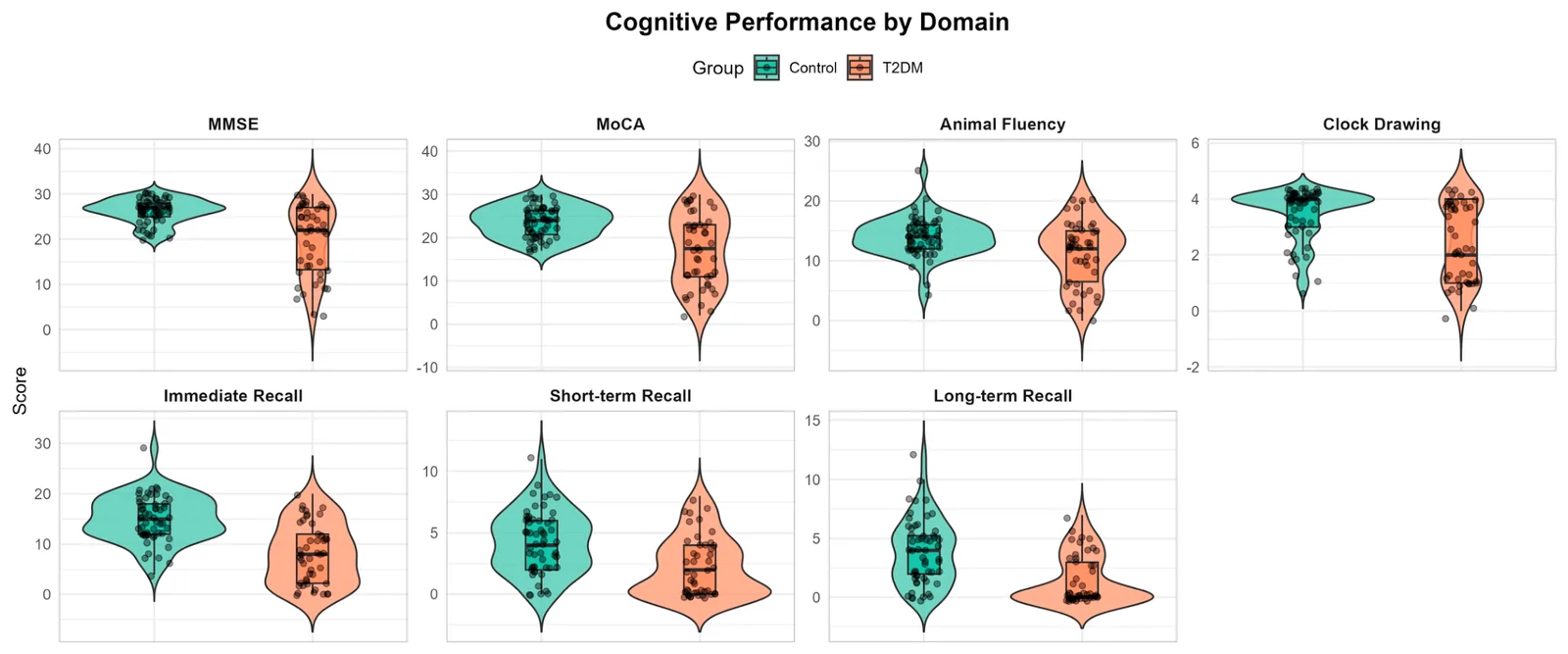
Cognitive performance by domain in T2DM versus controls. Violin plots (with width reflecting data density) with overlaid boxplots and individual data points show score distributions across seven cognitive domains. The T2DM group (orange) exhibited significantly lower performance than controls (green) in all domains (all *p* < 0.01), with the largest effect sizes observed for immediate recall, MMSE, and MoCA (all *p* < 0.001). Violin width reflects data density.

Patients with T2DM performed worse than non-diabetic controls in global cognition, specific cognitive domains, daily function, and physical performance (Table 2). MMSE and MoCA scores (mean ± SD) were lower in the T2DM group than in non-diabetic controls (MMSE: 19.76 ± 7.85 vs. 26.18 ± 2.70; MoCA: 17.15 ± 8.30 vs. 23.62 ± 3.61; both *p* < 0.001). Scores (mean ± SD) on animal fluency (11.28 ± 5.34 vs. 14.02 ± 3.28, *p* = 0.003) and clock drawing (2.46 ± 1.41 vs. 3.46 ± 0.89, *p* < 0.001) were also lower. Memory performance (mean ± SD) was reduced across immediate (8.07 ± 5.96 vs. 14.95 ± 4.59), short-term (2.26 ± 2.44 vs. 4.25 ± 2.54), and long-term recall (1.52 ± 2.09 vs. 3.80 ± 2.77) (all *p* < 0.001) in patients with T2DM compared to non-diabetic controls. Recruitment from a geriatric outpatient clinic produced broad cognitive performance ranges and large within-group variability (Figure 1). Table 2 presents descriptive, unadjusted comparisons; education was included as a covariate in all subsequent mediation analyses.

**Figure 2:**
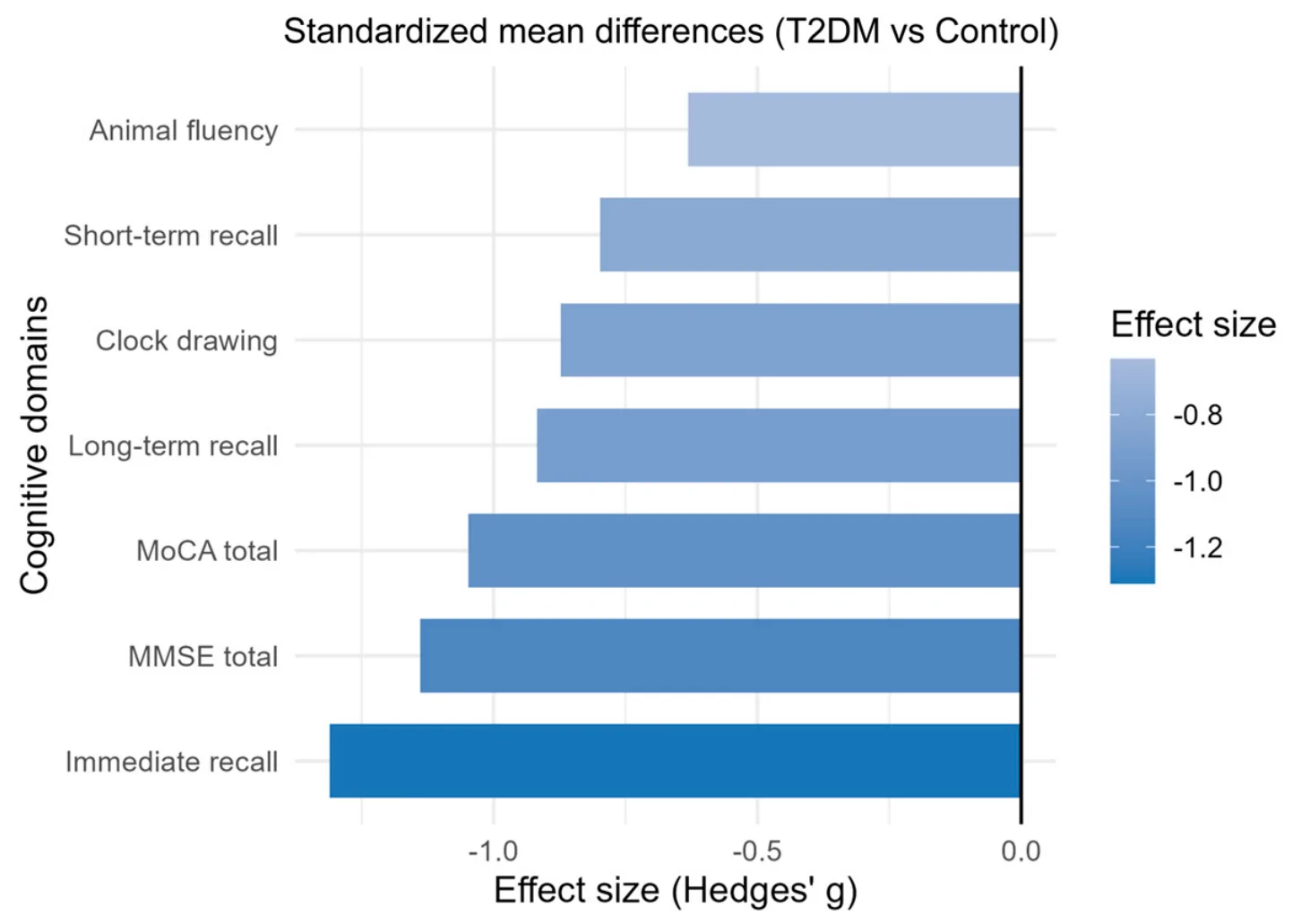
Standardized mean differences (Hedges’ g) in cognitive domains between T2DM and control groups. Negative values indicate worse performance in the T2DM group. Error bars represent 95% confidence intervals. Effect sizes are color-coded, with darker shades indicating larger impairments. All cognitive domains showed medium to very large effect sizes, with immediate recall, MMSE, and MoCA total scores demonstrating the greatest impairment (Hedges’ g < −1.0).

Standardized mean differences (Hedges’ g) indicated medium to very large effects for all cognitive measures (Table 3, Figure 2). The largest effects were observed for immediate recall (g = −1.31, 95% CI: −1.74 to −0.88), MMSE (g = −1.14, 95% CI: −1.56 to −0.72), and MoCA (g = −1.05, 95% CI: −1.46 to −0.63). Long-term recall (g = −0.92), clock drawing (g = −0.87), and short-term recall (g = −0.80) also showed large effects. However, the animal fluency CI was relatively wide, spanning small-to-large effects (g = −0.63).

There were no significant group differences in anxiety or depressive symptoms (HAMA: *p* = 0.709; HAMD: *p* = 0.637). Modestly higher IADL scores (median (IQR)) in T2DM participants suggest poorer instrumental functioning that may be clinically relevant in older adults (13.50 (12.00, 18.50) vs. 12.00 (12.00, 13.00), *p* = 0.003). Gait speed (mean ± SD) was slower in patients with T2DM than in non-diabetic controls (50.16 ± 15.04 vs. 78.49 ± 16.44 cm/s, *p* < 0.001) and grip strength (mean ± SD) was lower in patients with T2DM than in non-diabetic controls (19.35 ± 8.59 vs. 24.71 ± 7.00 kg, *p* = 0.001).

### 3.3. Correlations Between Cognitive Function and Physical Performance

**Figure 3:**
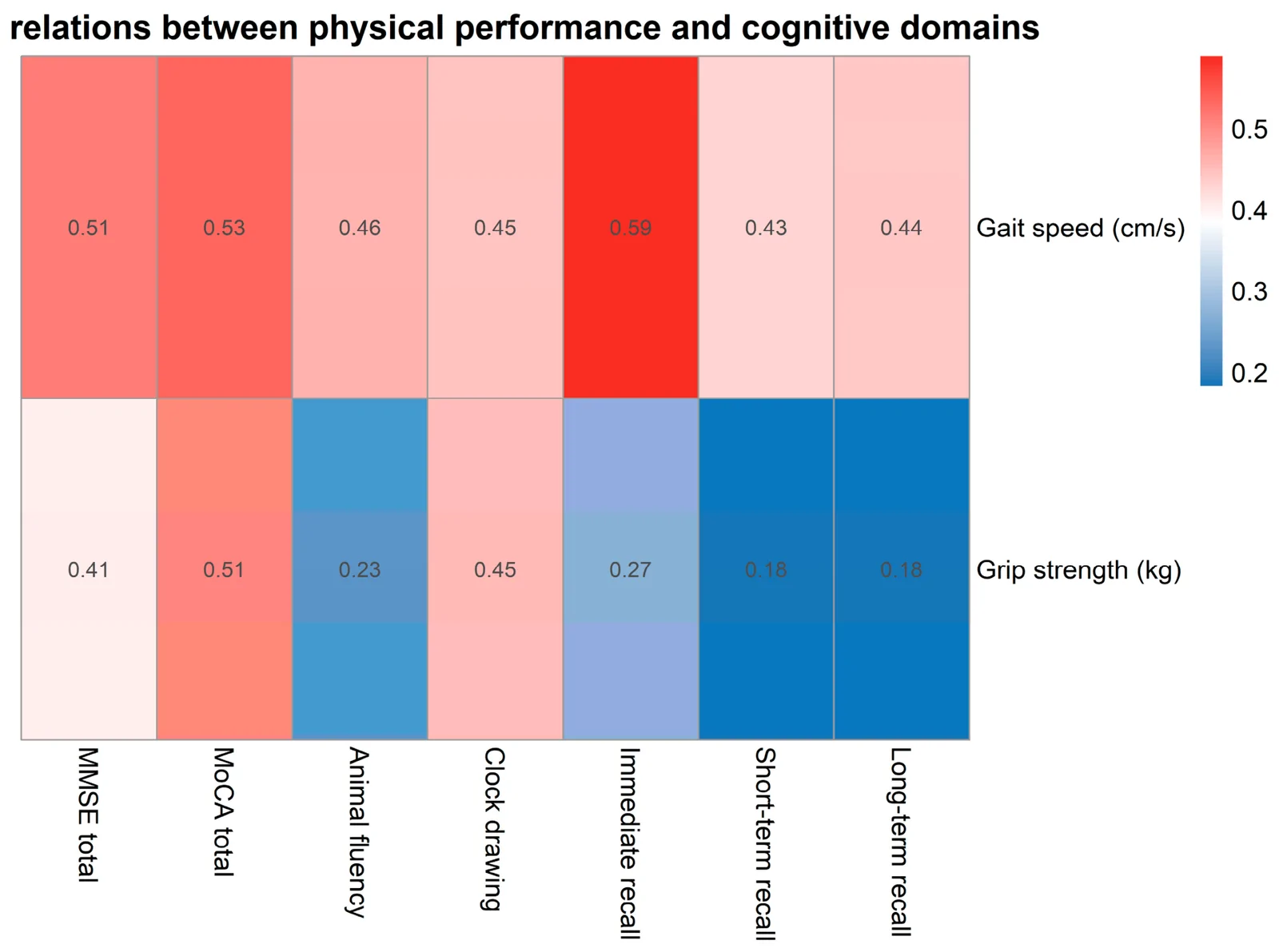
Heatmap of correlations between physical performance and cognitive domains within the T2DM group. Values represent Pearson correlation coefficients (r). Red shades indicate positive correlations, with darker red representing stronger associations; blue shades indicate weaker or non-significant correlations. Gait speed showed consistent moderate-to-strong correlations with all cognitive domains (all r ≥ 0.43, *p* < 0.001), with the strongest association observed for immediate recall (r = 0.59). Grip strength demonstrated weaker correlations overall, with non-significant associations observed for short-term and long-term recall (both r = 0.18, *p* = 0.065).

Correlation analyses were restricted to the T2DM group because the primary objective was to examine the relationship between cognitive and physical performance within individuals with diabetes. Correlations between cognitive measures and physical performance are presented in Table 4 and Figure 3. Gait speed was positively correlated with all cognitive indices. The strongest associations were with immediate recall (r = 0.59, *p* < 0.001), MoCA (r = 0.53, *p* < 0.001), and MMSE (r = 0.51, *p* < 0.001), with additional moderate correlations for animal fluency (r = 0.46), clock drawing (r = 0.45), long-term recall (r = 0.44), and short-term recall (r = 0.43) (all *p* < 0.001).

Grip strength also showed positive correlations with most cognitive measures, though generally weaker than those for gait speed. Significant correlations were found with MoCA (r = 0.51, *p* < 0.001), clock drawing (r = 0.45, *p* < 0.001), MMSE (r = 0.41, *p* < 0.001), immediate recall (r = 0.27, *p* = 0.006), and animal fluency (r = 0.23, *p* = 0.021). Correlations with short-term and long-term recall were relatively weak (both r = 0.18, *p* > 0.05), possibly requiring a larger sample size.

### 3.4. Mediation of the Association Between T2DM and Physical Performance by Global Cognition

Causal mediation analyses were conducted to examine whether global cognition (MoCA total score) mediated the association between T2DM and physical performance, adjusting for age, sex, years of education, BMI, and hypertension (Table 5).

For gait speed, the total effect of T2DM was significant (β = −28.423 cm/s, 95% CI: −34.259 to −22.597, *p* < 0.001). This consisted of a direct effect (ADE) of −23.232 cm/s (95% CI: −29.437 to −16.655, *p* < 0.001) and an indirect effect through MoCA (ACME) of −5.191 cm/s (95% CI: −8.728 to −2.320, *p* < 0.001). The proportion mediated was 18% (95% CI: 8–32%, *p* < 0.001).

For grip strength, the total effect of T2DM was also significant (β = −3.593 kg, 95% CI: −6.283 to −0.938, *p* = 0.010). The indirect effect via MoCA was −2.159 kg (95% CI: −3.780 to −0.809, *p* = 0.002), whereas the direct effect was smaller and not significant (ADE = −1.435 kg, 95% CI: −4.043 to 1.191, *p* = 0.282). Although the proportion mediated was 60% (95% CI: 23–181%, *p* = 0.012), the wide CI warrants cautious interpretation.

These results suggest that global cognition statistically accounted for part of the observed association between T2DM and gait speed. For grip strength, the significant indirect effect and non-significant direct effect are consistent with an indirect association through global cognition, but the proportion mediated should be interpreted cautiously.

### 3.5. Sensitivity Analyses

To evaluate the robustness of the mediation findings, two complementary sensitivity analyses were performed. First, mediation models adjusted only for age and sex yielded effect estimates that were consistent with those of the fully adjusted models, indicating that additional adjustment for education, BMI, and hypertension did not materially alter the observed indirect effects (Supplementary Table S1).

Second, sensitivity analyses were conducted to assess the influence of potential unmeasured mediator–outcome confounding using the medsens procedure (Supplementary Figures S1 and S2). For the gait-speed model, the estimated average mediation effect (ACME) remained different from zero until the residual correlation parameter reached approximately ρ = 0.35, indicating moderate robustness to potential unmeasured confounding. Similarly, for grip strength, the ACME approached zero at approximately ρ = 0.40, suggesting that a relatively strong residual confounder would be required to completely explain the observed indirect effect.

## 4. Discussion

Our study provides novel insights into the interaction between cognitive function and physical performance in the elderly population with T2DM recruited from outpatient clinics. Compared with age-matched non-diabetic controls, T2DM individuals showed significant impairments in multiple cognitive domains and physical performance indicators. Specifically, T2DM participants scored significantly lower in the overall cognitive assessment (MMSE and MoCA). At the same time, there were deficits in immediate recall, short-term recall, long-term recall, animal fluency, and clock drawing performance. Physical performance was also significantly impaired. Compared with the controls, the gait speed of T2DM individuals was significantly slower and the grip strength was lower. Importantly, cognitive function and physical performance indicators were moderately correlated, suggesting that there was an interrelated decline in these two domains. In this exploratory cross-sectional analysis, statistical mediation models suggested that variation in MoCA total score accounted for part of the association between T2DM status and physical performance. Shared T2DM-related mechanisms may underlie declines in both cognition and mobility. These findings should be interpreted as hypothesis-generating rather than evidence of a causal pathway.

The cognitive impairments we observed in the T2DM cohort are consistent with the multi-domain cognitive defects of diabetics recorded in the substantial amount of literature. Recent meta-analytic evidence shows that T2DM diagnosed in adulthood will significantly impair memory and overall cognition, and these defects will persist and be more obvious in later life [26]. Our findings add further evidence in support of these patterns. Patients with T2DM have significant impairments in overall cognition, immediate and delayed recall, and attention. The magnitude of the cognitive differences between the two groups suggests substantial cognitive impairment in this outpatient sample, although MoCA is a screening instrument and should not be interpreted as a diagnostic measure of dementia. Particularly prominent defects in immediate recall may reflect the vulnerability of the hippocampal-dependent episodic memory coding process, which may be compromised by hippocampal atrophy and cerebrovascular pathology induced by chronic hyperglycemia [27,28]. Because the present study did not include comprehensive neuropsychological testing or neuroimaging, mechanistic explanations regarding specific neural circuits remain speculative. Taken together, prior evidence and our findings suggest that cognitive decline in T2DM is a widespread impairment pattern that affects a variety of neural systems and may have an impact on daily function and quality of life.

In parallel with cognitive impairment, our study also documented obvious physical performance deficits in elderly patients with T2DM. Compared with the controls, both gait speed and grip strength of the diabetic group were significantly reduced. The gait speed is widely regarded as a vital sign of the overall health status of the older adults, of which a gait speed below 0.6 m/s is associated with a higher risk of falls, hospitalization, disability and death [29]. Similarly, diminished grip strength is a sign of systemic muscle weakness and has been regarded as a key criterion for sarcopenia and frailty, and both can predict functional decline and loss of independence [30]. The mechanism of physical performance deficits in T2DM is multi-factorial, including diabetic peripheral neuropathy that impairs proprioception and motor control; accelerated sarcopenia driven by chronic inflammation and insulin resistance; microvascular complications affecting muscle perfusion; and mitochondrial dysfunction that reduces muscle energy metabolism [31,32]. The relatively large direct effect observed for gait speed in the mediation model further suggests that peripheral neuropathy, sarcopenia, vascular dysfunction, and other diabetes-related complications probably contribute substantially to mobility impairment independently of cognition.

An important finding of this study is that cognitive function was associated with physical performance in older adults with T2DM. Because correlation analyses were restricted to participants with T2DM, these findings primarily reflect within-diabetes variability rather than differences between diabetic and non-diabetic individuals. Correlation analyses showed that there was a moderate correlation between cognitive indicators and physical performance indicators. The modest effect sizes likely reflect the multifactorial determinants of cognition and mobility and the restricted clinical range due to eligibility criteria, which may attenuate associations. The correlations between immediate memory and gait speed and between overall cognition and gait speed and grip strength were particularly prominent. These results suggest that in T2DM, cognition and physical function do not decline independently of each other, but show a parallel and interconnected deterioration. The interaction between cognition and physical decline may reflect impaired sensory-motor integration. Individuals with “dual decline” show greater volume loss in frontal, parietal, subcortical, and cerebellar areas, alongside accelerated atrophy in medial temporal, parietotemporal, and perisylvian regions—areas critical for memory encoding, motor control, and sensorimotor integration [33,34]. Gait, especially in challenging environments, requires continuous cognitive monitoring, attention, executive planning, and spatial working memory. When the memory system is damaged, these processes may be affected [35]. In addition to these task-related cognitive demands, the correlation we observed may also reflect the common pathophysiological mechanisms affecting the brain and muscle tissue in T2DM. Chronic hyperglycemia, insulin resistance, systemic inflammation and microvascular damage can compromise nerve integrity and skeletal muscle function at the same time, thus forming a coordinated decline pattern across cognitive and physical fields [36,37]. These findings add to the literature suggesting that cognitive and physical functions may decline in parallel in older adults with T2DM.

The most important contribution of this study is to provide exploratory evidence suggesting that poorer global cognitive performance may account for part of the observed association between T2DM and physical performance. Although the indirect effect for grip strength was statistically significant, the proportion mediated had a wide confidence interval extending beyond 100%, indicating considerable statistical uncertainty. Therefore, interpretation should focus primarily on the indirect effect rather than the proportion mediated. Different mediation patterns may reflect differences in the relative contribution of cognitive and non-cognitive mechanisms across physical performance outcomes. The larger indirect effect observed for grip strength may reflect the higher cognitive needs of maximum muscle contraction movement for sustained attention and motor planning; however, this interpretation is speculative and needs to be tested. Meanwhile, the decrease in gait speed may reflect central cognitive factors and peripheral neuromuscular pathology, including diabetic neuropathy and sarcopenia. These associations are consistent with shared pathophysiological mechanisms operating through the “muscle–brain axis”. This axis is a two-way communication network that is increasingly considered an important link between sarcopenia and cognitive decline in T2DM. Chronic hyperglycemia can induce endothelial dysfunction and microvascular damage, thus impairing brain perfusion and skeletal muscle microcirculation at the same time [38,39]. T2DM also has the characteristics of chronic low-grade inflammation and mitochondrial dysfunction, forming a systematic environment in which pro-inflammatory cytokines and oxidative stress promote both neuroinflammation and muscle catabolism [40]. Importantly, disrupted myokine secretion, including the reduction in neuroprotective factors such as brain-derived neurotrophic factors (BDNF), irisin and insulin-like growth factor-1 (IGF-1) may represent one of several biologically plausible hypotheses, because these exercise-induced cytokines normally promote both myogenesis and synaptic plasticity [41]. Because these biomarkers were not measured in the present study, these mechanisms remain speculative. Nevertheless, exercise trials show that a combined aerobic–resistance program can modulate adipokines (e.g., leptin and adiponectin) and inflammatory markers (e.g., IL-6, TNF-α, and IL-17A) alongside improvements in body composition, providing a plausible context for an inflammation/adipokine-related muscle–brain framework [42]. In addition, diabetic cerebrovascular diseases that affect the motor cortex and corticospinal tract integrity may directly damage neural drive to muscles, resulting in weakness independent of peripheral pathology [43]. Therefore, the assessment of older adults with T2DM should integrate cognitive and physical evaluation, because defects in one domain may be associated with damage to another area. From a clinical perspective, these findings do not imply that cognition causes physical decline. Rather, poorer cognitive performance may serve as an early clinical marker identifying individuals with T2DM who are also at increased risk of mobility impairment. If confirmed in longitudinal studies, combined cognitive and physical assessment may help identify older adults with T2DM who could benefit from multidomain interventions before overt mobility disability develops [44]. Notably, cognitive performance accounted for a larger proportion of the observed association for grip strength than for gait speed; however, this proportion is a ratio and can be imprecise when confidence intervals are wide, so this difference should be interpreted cautiously. Gait speed and grip strength may reflect partially different physiological determinants, which may contribute to differing correlation patterns across outcomes. The consistency of the minimally adjusted mediation models and the moderate robustness observed in the mediation sensitivity analyses further support the stability of the observed indirect effects, although they cannot eliminate residual confounding inherent to cross-sectional mediation analyses.

This study has several limitations. First, the cross-sectional design precludes causal inference or confirmation of temporal ordering between T2DM, cognition, and physical performance. Second, the relatively modest sample size may have limited precision, particularly for the grip-strength mediation model. Third, diabetes duration, HbA1c, and complication burden were unavailable and residual confounding cannot be excluded. Fourth, MoCA is a screening instrument rather than a comprehensive neuropsychological assessment and therefore may not fully capture domain-specific cognitive dysfunction. Finally, the extensive exclusion criteria and recruitment from a geriatric outpatient clinic may limit the generalizability of these findings.

## 5. Conclusions

In this exploratory cross-sectional study, MoCA total score statistically accounted for part of the association between T2DM and physical performance. These findings support the hypothesis that cognitive and physical impairment may represent interconnected manifestations of diabetes-related aging rather than establishing a causal mediating pathway. If validated in longitudinal studies, integrated cognitive and physical assessment may facilitate earlier identification of older adults with T2DM at increased risk of functional decline.

## Figures and Tables

**Table 1:** Baseline characteristics of T2DM and control groups.

Variable	Category	Control (CON)	T2DM	*p* Value
Continuous variables				
Age (years)		73.48 ± 7.47	74.72 ± 8.04	0.427
Education (years)		9.91 ± 4.19	7.87 ± 5.26	0.036
BMI (kg/m^2^)		22.45 (20.25, 24.80)	22.50 (20.25, 24.70)	0.649
Categorical variables				
Sex	female	23 (41.1%)	25 (54.3%)	0.255
	male	33 (58.9%)	21 (45.7%)	
Hypertension	no	36 (64.3%)	21 (45.7%)	0.092
	yes	20 (35.7%)	25 (54.3%)	

**Table 2:** Comparison of cognitive, affective, and daily function and physical performance between T2DM and control groups.

Domain	Measure	CON	T2DM	*p* Value
Global cognition	MMSE total score	26.18 ± 2.70	19.76 ± 7.85	<0.001
	MoCA total score	23.62 ± 3.61	17.15 ± 8.30	<0.001
Specific cognitive domains	Animal fluency test	14.02 ± 3.28	11.28 ± 5.34	0.003
	Clock drawing test	3.46 ± 0.89	2.46 ± 1.41	<0.001
	Immediate recall	14.95 ± 4.59	8.07 ± 5.96	<0.001
	Short-term recall	4.25 ± 2.54	2.26 ± 2.44	<0.001
	Long-term recall	3.80 ± 2.77	1.52 ± 2.09	<0.001
Affective symptoms	HAMA anxiety score	3.00 (1.00, 4.25)	2.00 (0.00, 5.00)	0.709
	HAMD depression score	2.00 (0.00, 4.25)	1.00 (0.00, 4.00)	0.637
Daily function and physical performance	IADL score	12.00 (12.00, 13.00)	13.50 (12.00, 18.50)	0.003
	Gait speed (cm/s)	78.49 ± 16.44	50.16 ± 15.04	<0.001
	Grip strength (kg)	24.71 ± 7.00	19.35 ± 8.59	0.001

**Table 3:** Standardized mean differences in cognitive domains between T2DM and control groups.

Cognitive Domain	CON	T2DM	Effect Size (Hedges’ g) [95% CI]
Immediate recall	14.95 ± 4.59	8.07 ± 5.96	−1.31 (−1.74, −0.88)
MMSE total	26.18 ± 2.70	19.76 ± 7.85	−1.14 (−1.56, −0.72)
MoCA total	23.62 ± 3.61	17.15 ± 8.30	−1.05 (−1.46, −0.63)
Long-term recall	3.80 ± 2.77	1.52 ± 2.09	−0.92 (−1.33, −0.51)
Clock drawing	3.46 ± 0.89	2.46 ± 1.41	−0.87 (−1.28, −0.46)
Short-term recall	4.25 ± 2.54	2.26 ± 2.44	−0.80 (−1.20, −0.39)
Animal fluency	14.02 ± 3.28	11.28 ± 5.34	−0.63 (−1.03, −0.23)

**Table 4:** Correlations between physical performance and cognitive domains in participants with T2DM.

Physical Performance	MMSE Total	MoCA Total	Animal Fluency	Clock Drawing	Immediate Recall	Short-Term Recall	Long-Term Recall
Gait speed (cm/s)	0.51 (<0.001)	0.53 (<0.001)	0.46 (<0.001)	0.45 (<0.001)	0.59 (<0.001)	0.43 (<0.001)	0.44 (<0.001)
Grip strength (kg)	0.41 (<0.001)	0.51 (<0.001)	0.23 (0.021)	0.45 (<0.001)	0.27 (0.006)	0.18 (0.065)	0.18 (0.065)

**Table 5:** Mediation of the association between T2DM and physical performance by global cognition (MoCA). ACME, average causal mediation effect (indirect effect); ADE, average direct effect. Models are adjusted for age, sex, years of education, body mass index (BMI), and hypertension.

Outcome	Effect Type	Estimate (95% CI)	*p*-Value
Gait speed (cm/s)	ACME (indirect effect)	−5.191 (−8.728, −2.320)	<0.001
Gait speed (cm/s)	ADE (direct effect)	−23.232 (−29.437, −16.655)	<0.001
Gait speed (cm/s)	Total effect	−28.423 (−34.259, −22.597)	<0.001
Gait speed (cm/s)	Proportion mediated	0.18 (0.08, 0.32)	<0.001
Grip strength (kg)	ACME (indirect effect)	−2.159 (−3.780, −0.809)	0.002
Grip strength (kg)	ADE (direct effect)	−1.435 (−4.043, 1.191)	0.282
Grip strength (kg)	Total effect	−3.593 (−6.283, −0.938)	0.010
Grip strength (kg)	Proportion mediated	0.60 (0.23, 1.81)	0.012

## Data Availability

The data presented in this study are available on request from the corresponding author due to patient privacy protection and local medical data management regulations.

## Supplementary Materials

The following supporting information can be downloaded at https://www.mdpi.com/article/10.3390/healthcare14162581/s1: Table S1. Sensitivity analysis of the mediation models using minimally adjusted covariates (age and sex only). Figure S1. Sensitivity analysis for the indirect effect of MoCA on the association between T2DM and gait speed. Figure S2. Sensitivity analysis for the indirect effect of MoCA on the association between T2DM and grip strength.

## Abbreviations

The following abbreviations are used in this manuscript:

T2DM	Type 2 diabetes mellitus
MMSE	Mini-Mental State Examination
MoCA	Montreal Cognitive Assessment
MoCA-BJ	Beijing version of the Montreal Cognitive Assessment
AVLT	Auditory Verbal Learning Test
CDT	Clock Drawing Test
IADL	Instrumental Activities of Daily Living
BMI	Body mass index
HAMA	Hamilton Anxiety Rating Scale
HAMD	Hamilton Depression Rating Scale
ACME	Average causal mediation effect
ADE	Average direct effect
FDR	False discovery rate
BDNF	Brain-derived neurotrophic factor
IGF-1	Insulin-like growth factor-1
