# Supplementary material for: Cognitive Function Mediates the Association Between Type 2 Diabetes and Physical Performance Decline in Older Adults: A Cross-Sectional Study
Source: Healthcare (Basel). 2026 Aug 17;14(16):2581. doi: 10.3390/healthcare14162581 (PMC13512707; doi:10.3390/healthcare14162581)
Supplement: Supplementary file 1 [file healthcare-14-02581-s001.zip › healthcare-4454205-supplementary.pdf]

Supplementary Table S1. Sensitivity analysis of the mediation models using minimally adjusted covariates (age and sex only).

Outcome	ACME (95% CI)	ADE (95% CI)	Total Effect (95% CI)	Proportion Mediated (95% CI)	P value
**Gait speed**	−4.07 (−7.58, −1.46)	−23.69 (−30.13, −17.18)	−27.76 (−33.58, −21.80)	0.147 (0.052–0.285)	<0.001
**Grip strength**	−2.39 (−3.91, −1.06)	−1.42 (−3.86, 1.05)	−3.82 (−6.18, −1.48)	0.627 (0.274–1.623)	<0.001

Table footnote

Abbreviations: ACME, average causal mediation effect; ADE, average direct effect; CI, confidence interval.

Mediation analyses were repeated using models adjusted for age and sex only to examine the robustness of the primary findings. Estimates were obtained using 5000 nonparametric bootstrap resamples.

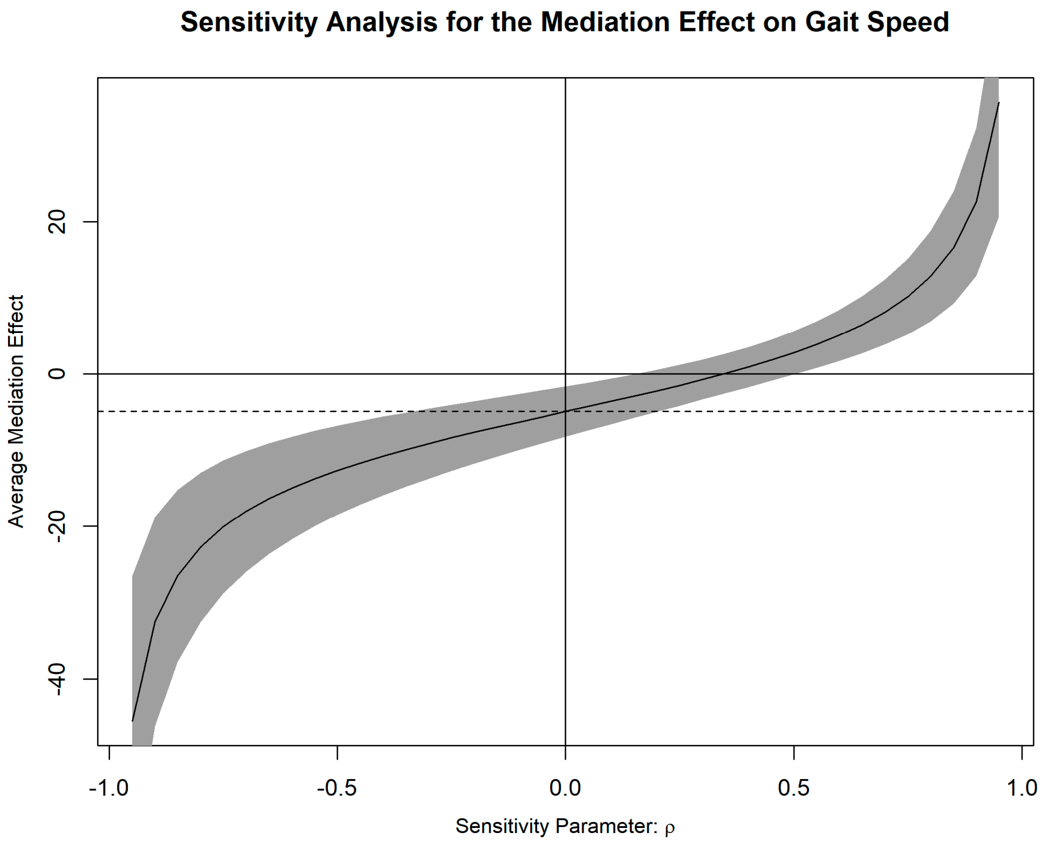

Supplementary Figure S1. Sensitivity analysis for the indirect effect of MoCA on the association between T2DM and gait speed. The solid curve represents the estimated average causal mediation effect (ACME) across increasing values of the sensitivity parameter (ρ), which reflects the correlation between residuals of the mediator and outcome models attributable to hypothetical unmeasured confounding. The shaded region indicates the 95% confidence interval. The estimated ACME crossed zero at approximately **ρ = 0.35**, suggesting moderate robustness of the indirect effect to potential unmeasured mediator–outcome confounding.

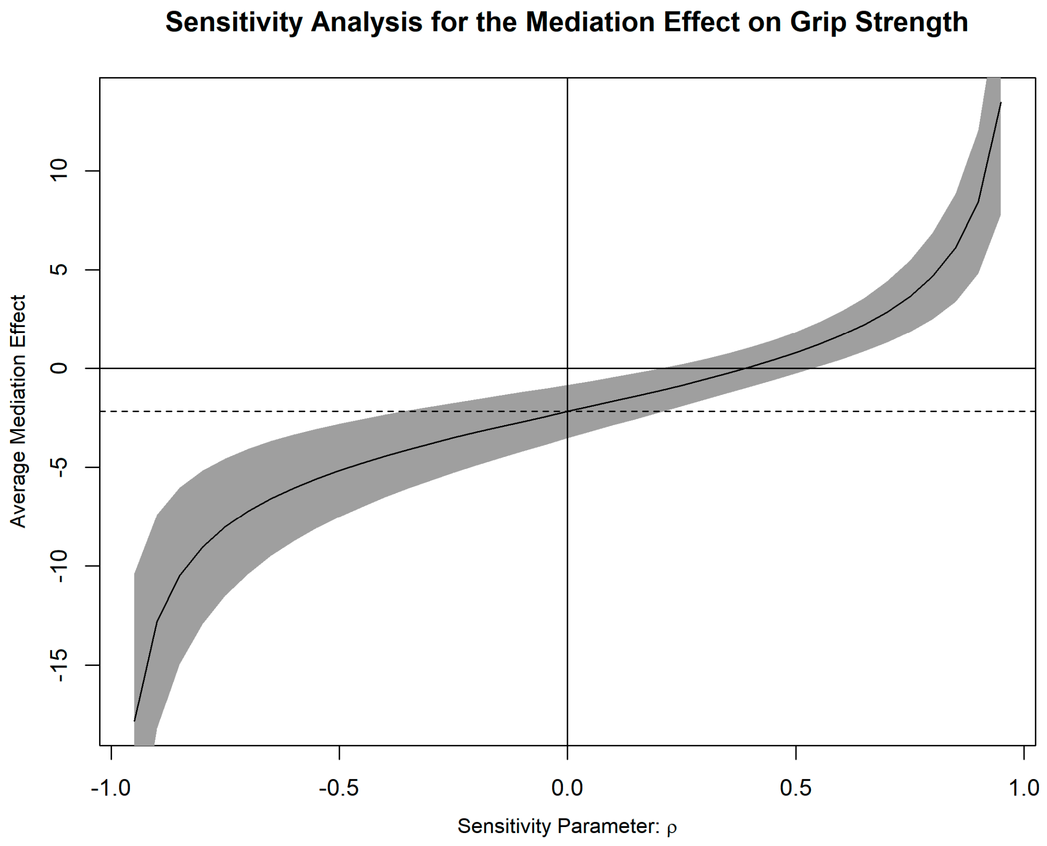

Supplementary Figure S2. Sensitivity analysis for the indirect effect of MoCA on the association between T2DM and grip strength. The solid curve represents the estimated average causal mediation effect (ACME) across increasing values of the sensitivity parameter (ρ). The shaded region represents the 95% confidence interval. The estimated ACME approached zero at approximately **ρ = 0.40**, indicating that the observed mediation effect remained relatively robust unless moderate residual confounding between the mediator and outcome was present.
